# Assessing validity of a short food frequency questionnaire on present dietary intake of elderly Icelanders

**DOI:** 10.1186/1475-2891-11-12

**Published:** 2012-03-13

**Authors:** Tinna Eysteinsdottir, Inga Thorsdottir, Ingibjorg Gunnarsdottir, Laufey Steingrimsdottir

**Affiliations:** 1 Unit for Nutrition Research, University of Iceland and Landspitali National-University Hospital, Reykjavik, Iceland; 2 Faculty of Food Science and Human Nutrition, School of Health Sciences, University of Iceland, Reykjavik, Iceland; 3 Unit for Nutrition Research, Landspitali-University Hospital, Eiriksgata 29, IS-101 Reykjavik, Iceland

**Keywords:** Food frequency questionnaire, Validity, Elderly, Nutrition

## Abstract

**Background:**

Few studies exist on the validity of food frequency questionnaires (FFQs) administered to elderly people. The aim of this study was to assess the validity of a short FFQ on present dietary intake, developed specially for the AGES-Reykjavik Study, which includes 5,764 elderly individuals. Assessing the validity of FFQs is essential before they are used in studies on diet-related disease risk and health outcomes.

**Method:**

128 healthy elderly participants (74 y ± 5.7; 58.6% female) answered the AGES-FFQ, and subsequently filled out a 3-day weighed food record. Validity of the AGES-FFQ was assessed by comparing its answers to the dietary data obtained from the weighed food records, using Spearman's rank correlation, Chi-Square/Kendall's tau, and a Jonckheere-Terpstra test for trend.

**Result:**

For men a correlation ≥ 0.4 was found for potatoes, fresh fruits, oatmeal/muesli, cakes/cookies, candy, dairy products, milk, pure fruit juice, cod liver oil, coffee, tea and sugar in coffee/tea (r = 0.40-0.71). A lower, but acceptable, correlation was also found for raw vegetables (r = 0.33). The highest correlation for women was found for consumption of rye bread, oatmeal/muesli, raw vegetables, candy, dairy products, milk, pure fruit juice, cod liver oil, coffee and tea (r = 0.40-0.61). An acceptable correlation was also found for fish topping/salad, fresh fruit, blood/liver sausage, whole-wheat bread, and sugar in coffee/tea (r = 0.28-0.37). Questions on meat/fish meals, cooked vegetables and soft drinks did not show a significant correlation to the reference method. Pearson Chi-Square and Kendall's tau showed similar results, as did the Jonckheere-Terpstra trend test.

**Conclusion:**

A majority of the questions in the AGES-FFQ had an acceptable correlation and may be used to rank individuals according to their level of intake of several important foods/food groups. The AGES-FFQ on present diet may therefore be used to study the relationship between consumption of several specific foods/food groups and various health-related endpoints gathered in the AGES-Reykjavik Study.

## Background

Food frequency questionnaires (FFQ) are important research tools in nutritional epidemiology, and assessing their validity is an essential prerequisite for their use in studies of diet-related disease risk [1,2]. Few studies exist on the validity of FFQs administered to elderly people [3-5], and many of the instruments used were originally developed for younger subjects. Hence, their reliability and validity when administered to older subjects is uncertain [6,7].

It is always a challenge to assess dietary intake, and perhaps even more so when elderly individuals are concerned. Various factors related to older age, such as fading memory, declined cognitive function, and impaired hearing and/or vision may possibly affect the ability to give reliable information on dietary intake [4-10]. It has been suggested that FFQs may be a more appropriate assessment method for older people than, for example, 24 hour recalls [5,6] as older individuals may have more problems with short-term than long-term recalls, as well as more difficulties with open-ended recalls than with structured ones [10]. The length of interviews and questionnaires is crucial as older people may require longer to answer and may become more fatigued and frustrated than younger people [5]. Long and extensive FFQs may therefore contribute to lower response rates among elderly people [4].

Weighed food records are widely used and accepted as an appropriate reference method when validating FFQs [11,12]. In spite of inherent weaknesses of any dietary assessment method, food records have often been considered as the "gold standard" as they can provide relatively accurate quantitative information on consumption. Elderly participants have proved to be capable of keeping food records with acceptable levels of compliance and completion [13], and food records have been found to provide valid intake data for free-living elderly individuals [14]. Generally, a food record consisting of 3-4 consecutive days is recommended, as studies have shown that incomplete records get more frequent as the number of days increases. This is referred to as respondent fatigue [15,16].

While short FFQs lack the detail of longer questionnaires or food records, they have nevertheless been found to adequately assess the intake of specific foods and rank individuals with respect to selected nutrients [17-20]. The short food frequency questionnaire (AGES-FFQ) being assessed here was specially designed for the AGES-Reykjavik study, with 5,764 elderly participants. The AGES-Reykjavik study examines risk factors, genetic susceptibility, and gene/environment interaction, including diet, in relation to disease and disability in old age. Extensive health-related variables have been gathered for all participants. The AGES-Reykjavik study has been described previously [9]. The study was approved by the Icelandic National Bioethics Committee (VSN: 00-063) and the MedStar IRB for the Intramural Research Program, Baltimore, MD. The AGES-FFQ is threefold, including questions on diet in early life (14-19 y), midlife (40-50 y) and present diet. The validity of questions on midlife diet has been assessed in a previous study, where simple questions on consumption of, e.g., fish, meat, milk/dairy products, and cod liver oil were found to be valid [21].

The aim of this study is to assess the validity and ability of the AGES-FFQ to rank individuals according to intake of selected foods and food groups and to distinguish between individuals having high vs. low intake. Assessing the validity of the AGES-FFQ is essential before studying the relationship between present diet and health-related variables in the AGES-Reykjavik Study.

## Methods

### Subjects and setting

Subjects were healthy, elderly people, 65 years and older (58.6% female), and were a subsample of participants in the IceProQualita study, which focuses on the effects of training and food supplements on various health factors and health-related quality of life among the elderly [22]. Participants were recruited into the IceProQualita by advertisements posted in community centres and residential care homes in the capital area of Iceland. The advertisements included information on the study protocol and contact numbers. Willing and eligible individuals phoned in for further information and registration. A total of 284 individuals were registered and screened; 47 were excluded, leaving 237 participants at baseline. Exclusion criteria were cognitive function < 19 points on the MMSE [23], uncontrolled coronary heart disease, pharmacological interventions with exogenous testosterone or other drugs known to influence muscle mass, and major orthopaedic disease. Participant also had to be free of any musculoskeletal disorders, had to be weight stable and all women postmenopausal.

Our subsample consisted of the first 137 participants enrolled into the IceProQualita Study by March 2009, when data analysis for the present study began. By that time these individuals had undergone all baseline measurements, filled out a 3-day food record, the AGES-FFQ on present diet, and signed an informed written consent. The IceProQualita study was approved by the Icelandic National Bioethics Committee (VSNb2008060007/03-15). Dietary records from nine individuals were considered incomplete or inadequate and were therefore excluded. Data from 128 participants were therefore included in this study. The dropout rate from the IceProQualita study was 12% (n = 29), illness and falls being the most common reason [22]. The dropout did not affect participation in the present study, however, as all validation data were gathered at baseline.

Furthermore, our subsample did not differ from the whole study group of the IceProQualita study regarding age, anthropometric measurements, physical performance test, and outcome of various questionnaires on, e.g., general health, anxiety, quality of life, and the Mini Mental State Examination (MMSE).

### Design

All participants answered the AGES-FFQ and subsequently filled out a 3-day weighed food record within approximately two weeks. Participants also completed questionnaires on physical activity, health-related quality of life, and drug, vitamin and herbal medicine intake. Anthropometric measures were performed; body weight was measured in light underwear on a calibrated scale (model no. 708, Seca, Hamburg, Germany), and height was measured with a calibrated stadiometer (model no. 206; Seca, Hamburg, Germany) [22].

The AGES-FFQ was used to assess frequency of consumption of different foods and food groups in order to rank individuals according to their level of intake. Validity of the AGES-FFQ on present diet was assessed by comparing its answers to the dietary data obtained from the weighed food records.

### The weighed food record

Before filling out the food records each participant met with a researcher and was provided with a household scale (PHILIPS Essence HR 2393) and a structured booklet for recording his or her intake. Participants received detailed oral instructions on how to weigh and record their intake and were shown how to use the household scale. Written instructions were also incorporated in the food booklets along with contact information in case any questions arose during recording. Participants were asked to record in the booklet all food and beverages consumed for three consecutive days (Thursday-Saturday or Sunday-Tuesday), along with dates and times of meals. The importance of maintaining their regular diets and weighing and recording all food and drink consumed was emphasized.

### The food frequency questionnaire

The food frequency questionnaire was designed specifically for the AGES-Reykjavik Study and is divided into three parts, containing questions on early life diet (14-19 y), midlife diet (40-50 y) and present diet. The part of the questionnaire on present diet includes 30 questions, 21 of which are assessed here. These are questions on the average frequency of intake of major food groups, e.g., milk and dairy products, meat, fish, bread, fruits and vegetables, as well as questions on selected foods, such as rye bread, blood/liver sausage, oat meal porridge and cod liver oil. Foods and food groups were selected for the questionnaire on the basis of their contribution to the absolute intake of elderly Icelanders according to former National Nutrition Surveys, as well as their unique nutritional qualities and possible connection to the development of various diseases in later life. The remaining nine questions, not assessed in the present study, are on the frequency of hot meals, type of milk and dairy products most commonly used, type and amount of bread spread commonly used, and finally there are four questions related to salt consumption (perception of saltiness, consumption of salted meat, salt fish, and added salt to prepared meals).

**Figure 1:**
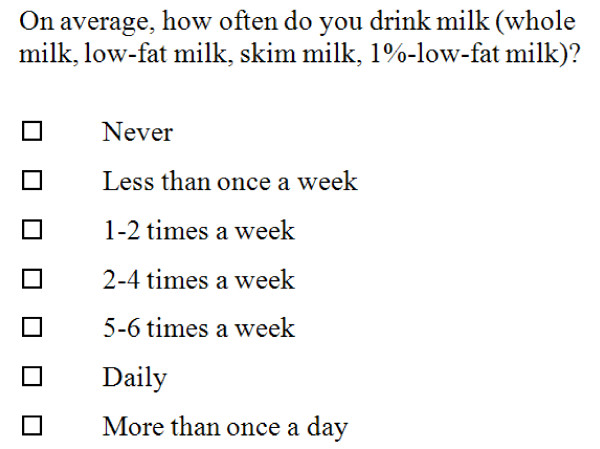
**Example of a question from the AGES-FFQ on present diet and response categories**.

A majority of the questions have the same possible response categories as the questionnaire was designed to be simple and easily completed by elderly individuals (Figure 1 shows an example of question and response categories from the AGES-FFQ). However, questions on coffee, tea and sugar in coffee/tea differed in that they asked about daily frequency rather than weekly frequency of consumption. The questions not assessed here also had different response categories related to types of products, such as low fat vs. high fat, and salt perception.

#### Data analysis

##### Nutritional analysis and data management

Data on the participants' intake according to the 3-day weighed food records were entered into an interview-based nutrient calculation program, ICEFOOD, designed for the national dietary survey of The Icelandic Nutrition Council [24]. The amount of foods/food groups was calculated from 452 food recipes, which are based on 1148 food items from the National Nutritional Database, ISGEM.

Individual intake in grams per day for each food/food group was calculated from the food records. Gender-specific portions were estimated taking into account actual intake in grams and eating occasions from the food diaries, as well as predetermined portion sizes used in our previous validation study of questions on midlife diet (Additional file 1) [21]. The gender-specific portions were used to calculate intake in grams from the AGES-FFQ. The correlation was calculated between grams of food intake according to the two methods, food records and the AGES-FFQ.

##### Statistical analysis

Data were entered into the statistical package SPSS, version 11.0. Kolmogorov-Smirnov tests were used to test the distribution of data. The answers from the AGES-FFQ were not normally distributed; neither were most of the data from the food records.

Simple descriptive statistics were used to describe general characteristics of the study group and the AGES participants. To assess differences between groups, student t-tests, Mann- Whitney U-test and Chi-square test were used. Correlation between intake according to the AGES-FFQ and the food records was assessed using Spearman's rank correlation. As the distribution of reported intake from the AGES-FFQ on present diet was skewed, subjects could not be divided into quartiles or quintiles. Data were therefore split into 2-4 groups, depending on the distribution of answers from each question of the AGES-FFQ. Kendall's tau-b rank correlation coefficient and Chi-Square tests were used to further examine association between the two methods.

Additionally, the computer program SAS version 9.1 was used to perform a nonparametric Jonckheere-Terpstra test for trend, to test if the categories according to the AGES-FFQ ranked mean intake from the food record in an anticipated, graded order.

The significance level was set at *p *≤ 0.05.

## Results

Comparing the AGES-FFQ to the reference method (Table 1), a correlation ≥ 0.4 was found for potatoes, fresh fruits, oatmeal/muesli, cakes/cookies, candy, dairy products, milk, pure fruit juice, cod liver oil, coffee, tea and sugar in coffee/tea (r = 0.40-0.71) for men. Furthermore, a correlation of 0.33 was found for raw vegetables. For women, correlation ≥ 0.4 was found for rye bread, oatmeal/muesli, raw vegetables, candy, dairy products, milk, pure fruit juice, coffee, tea and cod liver oil (r = 0.40-0.61). A correlation between 0.3 and 0.37 was found for fish topping/salad, fresh fruit, blood/liver sausage, and sugar in coffee/tea. The correlation for whole-wheat bread was lower, but still significant (r = 0.28, *p *= 0.017). Questions on meat and fish consumption, as well as questions on cooked vegetables and soft drinks, were not found to have a significant correlation to the reference method.

**Table 1:** Correlation between grams of intake from food records and calculated intake from the AGES-FFQ

	Food record g/d			AGES-FFQ g/d				
	P10	median	P90	P10	median	P90	correlation	p-value
**Men (n = 53)**								
Meat	7	90	248	43	100	100	0.21	0.124
Fish	20	77	190	36	85	114	0.23	0.098
Fish toppings	0	0	23	2	10	23	0.23	0.146
Potatoes	22	74	174	24	86	110	0.46	< 0.001
Fresh fruits	0	87	226	24	86	110	0.50	< 0.001
Blood/liver sausage	0	0	13	0	3	17	0.05	0.746
Rye bread/flatbread	0	17	59	2	25	50	0.17	0.219
Whole-wheat bread	11	45	73	11	50	50	0.19	0.169
Oatmeal/muesli	0	0	227	0	95	190	0.46	0.001
Cooked vegetables	0	29	113	3	19	60	0.17	0.221
Raw vegetables	0	33	150	3	19	90	0.33	0.015
Cakes and cookies	0	51	137	10	15	70	0.41	0.002
Candy	0	0	13	0	8	18	0.40	0.003
Dairy products	0	84	241	7	103	205	0.55	< 0.001
Milk	0	133	560	0	83	264	0.49	< 0.001
Pure fruit juice	0	0	192	0	34	160	0.50	< 0.001
Soft drink and sweet								
juice	0	0	134	0	12	231	0.19	0.177
Cod liver oil	0	0	8	0	6	6	0.51	< 0.001
Coffee*	87	357	793	105	735	1155	0.63	< 0.001
Tea*	0	0	200	0	110	330	0.71	< 0.001
Sugar in coffee/tea*	0	0	11	0	0	8	0.53	< 0.001
**Women (n = 75)**								
Meat	21	59	151	29	29	68	0.11	0.361
Fish	21	55	137	28	65	102	-0.02	0.873
Fish toppings	0	0	28	0	3	35	0.37	0.001
Potatoes	18	60	116	18	67	85	0.01	0.969
Fresh fruits	39	127	316	60	120	240	0.36	0.001
Blood/liver sausage	0	0	14	0	2	13	0.37	0.001
Rye bread/flatbread	0	7	40	2	30	60	0.42	< 0.001
Whole-wheat bread	1	35	76	10	45	45	0.28	0.017
Oatmeal/muesli	0	52	159	3	75	150	0.48	< 0.001
Cooked vegetables	0	25	90	3	45	90	0.20	0.089
Raw vegetables	0	53	141	17	40	80	0.40	< 0.001
Cakes and cookies	0	36	123	2	30	60	0.20	0.087
Candy	0	0	23	1	6	30	0.43	< 0.001
Dairy products	0	100	217	6	85	170	0.50	< 0.001
Milk	0	113	369	0	29	135	0.45	< 0.001
Pure fruit juice	0	0	167	0	34	160	0.49	< 0.001
Soft drink and sweet								
juice	0	0	148	0	8	48	0.19	0.104
Cod liver oil	0	2	9	0	7	7	0.42	< 0.001
Coffee*	67	283	647	98	293	683	0.44	< 0.001
Tea*	0	0	363	0	360	840	0.61	< 0.001
Sugar in coffee/tea*	0	0	3	0	0	0	0.30	0.008

* portions per day

The Jonckheere-Terpstra trend test gave comparable results to the Spearman's rank correlation, with the exception of fish topping/salad for men and cooked vegetables for women, which showed significant trend in spite of insignificant correlation (Additional file 2).

The Pearson Chi-Square and Kendall's tau gave similar results, also showing significant association for fish topping for men; however, no association was detected between the methods for consumption of raw vegetables and candy for men. Results for women were all comparable to the Spearman's rank correlation (Additional file 3).

General characteristics of participants in the present study are shown in Table 2, along with characteristics of the participants from the AGES-Reykjavik study, for which the AGES-FFQ was designed. Participants in the present study were on average slightly taller and significantly heavier, had a higher body mass index (BMI) and percent body fat than participants in AGES, and were on average 2.7 years younger than AGES participants.

**Table 2:** Comparison of study group and participants in the AGES-Reykjavik Study

	Men AGES	Study group	p-value	Women AGES	Study group	p-value
**Participants, n**	2102	53		2699	75	
**Age, y (sd)**	76.5 (5.3)	74.2 (6.0)	0.011	76.1 (5.5)	73.3 (5.5)	< 0.001
**Height, cm (sd)**	175.5 (6.2)	176.5 (7.1)	0.248	160.9 (5.7)	162.5 (5.7)	0.016
**Weight, kg (sd)**	82.6 (13.3)	92.7 (17.4)	< 0.001	70.5 (13.3)	74.8 (11.9)	0.004
**Smokers, %**	12	5.7	0.170	12.9	9.3	0.383
Physical activity, walk (sd)*	3.7 (3.0)	3.3 (4.0)	0.003	3.3 (3.7)	2.6 (2.6)	0.001
**Abdominal circumference, cm (sd)**	102.1 (10.5)	108.2 (12.7)	< 0.001	99.4 (12.9)	93.9 (11.2)	< 0.001
**BMI, kg/m**^**2 **^**(sd)**	26.8 (3.8)	29.7 (4.9)	< 0.001	27.2 (4.8)	28.4 (4.6)	0.035
**FFM, kg (sd)**	63.8 (7.6)	56.7 (6.9)	< 0.001	45.8 (6.3)	41.1 (4.6)	< 0.001
**FAT, kg (sd)**	18.5 (7.0)	32.9 (11.0)	< 0.001	24.3 (7.4)	31.7 (8.9)	< 0.001
**Percent body fat, % (sd)**	21.8 (5.5)	34.5 (5.9)	< 0.001	34.0 (5.0)	41.9 (6.6)	< 0.001
**Systolic blood pressure, mmHg (sd)**	142.8 (19.8)	148.0 (19.6)	0.005	141.9 (20.6)	137.2 (16.8)	0.060
**Diastolic blood pressure, mmHg (sd)**	76.0 (9.4)	77.6 (9.6)	0.357	72.1 (9.5)	74.1 (8.9)	0.099

*Hours spent walking per week, average time over the whole year

BMI = Body mass index; FFM = Fat free mass; FAT = Fat mass

## Discussion

The present study was conducted to assess validity of a dietary questionnaire and test its ability to rank individuals according to the level of intake of specific foods and food groups.

It has been suggested that when validating a questionnaire on present diet using a reference method, correlation coefficients should be ≥ 0.3 preferably over 0.4 and optimally in the range of 0.5-0.7 [11,12,25]. Of the 21 questions assessed here, 13 questions for the men and 14 for the women had a correlation ≥ 0.3 thereof 12 questions for the men and 10 for the women had a correlation ≥ 0.4 The foods showing the highest correlation were not in all cases identical for both genders, and men generally had higher correlations than women. The questions that had a correlation above 0.3 for both genders were on fresh fruits, oatmeal/muesli, raw vegetables, candy, dairy products, milk, pure fruit juice, cod liver oil, coffee, tea, and sugar used in coffee/tea.

The correlation between the AGES-FFQ and the reference method was not significant for fish, meat, cooked vegetables and soft drinks/sweetened juices. Part of the explanation for low or no correlation in general may be the inability of a 3-day food record to adequately reflect individual intake of foods that are consumed infrequently. Soft drinks are an example of this possible limitation of the reference method. For such food the 3-day food record may not be the ideal reference method, as the food in question may not show up on the food record. The AGES-FFQ data might even be closer to true intake in these cases. However, fish, meat and cooked vegetables were not consumed infrequently (2-4 times per week on average), and food items less frequently consumed had acceptable correlation between the two methods. A possible explanation for no correlation for meat and fish consumption might be the lack of distribution for answers to the AGES-FFQ, as almost 90% of participants marked either of two options - 1-2 times a week or 3-4 times a week - reflecting the uniform consumption of both fish and meat in this age group. Answers to the question on cooked vegetables were slightly better distributed even though almost 70% of participants answered either of the two previously mentioned options. In such cases, results from the food records may be better suited to rank individuals' intake. The validity of global questions with narrow distribution of answers, such as for meat, fish and vegetables, could presumably be improved by increasing frequency options to improve distribution, as well as by splitting them up into separate questions on types of meat, fish, etc. It is known that global questions may underestimate consumption [26], and affect validity. Global questions, chosen for the sake of simplicity, may thus limit the validity of the AGES-FFQ.

The results from the Jonckheere-Terpstra trend test, Kendall's tau-b and Pearson Chi-Square were mostly in agreement with the results from the Spearman's rank correlation, with few deviations. Limited and/or skewed distribution of answers from the AGES-FFQ may contribute to these differences between the methods.

The validity of questions on midlife diet (40-50 y) in the AGES-FFQ has previously been assessed [21]. Retrospective food intake was estimated, where elderly individuals answered the AGES-FFQ on midlife diet, and data were compared to a detailed dietary history, obtained from the same individuals 18-19 years previously, i.e., in midlife. Questions and frequency options were mostly similar for the two periods of life in the AGES-FFQ. In the validation study for midlife diet, the reference method may have been better able to detect correlation for food consumed < 2-3 times a week than the 3-day weighed food recording used in the present study. In the previous study a significant correlation was found for fish and meat consumption (r = 0.25-0.30), along with a stronger correlation for cod liver oil [21]. Part of the explanation for higher correlation in some cases might be linked to the detailed dietary history used as a reference method in the previous validation study, reflecting long-term diet. Another possible explanation for higher correlation for midlife diet might be that both dietary assessment methods, i.e., dietary history and FFQ, can be subject to similar sources of error, such as bias to overestimate foods considered healthy, and to underestimate foods considered unhealthy.

Looking at the distribution of intake according to the two methods, there was a tendency for higher consumption in grams from the food records than would be expected for certain foods/food groups, considering frequency of consumption according to the AGES-FFQ and the calculated consumption using gender-specific portions. This could partly be explained by exceptionally large portions consumed by a few individuals according to their food records. The largest single meat portion was 600 g; the largest portion of soda was 900 ml, and a few individuals had a daily consumption of milk ≥ 1000 ml, while their reported frequency of intake was 3-4 times per week to once a day. This discrepancy emphasizes the limitation of using an FFQ without portion sizes.

In an attempt to evaluate possible over-/underestimation of intake, frequency of intake was compared between the two methods, using actual eating occasions from the food records (data not shown). There was no clear sign of over-/underestimation related to gender or foods/food groups considered healthy/unhealthy. However, foods consumed infrequently according to the AGES-FFQ may not have shown up in the 3-day food records and lead to the perception of overestimation according to the AGES-FFQ. Reported frequency of milk intake according to the AGES-FFQ was generally lower than according to food records. One possible explanation may be that milk used in coffee/tea, or milk poured on breakfast cereals/porridge was not included when answering the AGES-FFQ.

In order to evaluate the representativeness of our study group, general characteristics of the group were compared to the participants of the AGES-Reykjavik study, for which the AGES-FFQ was designed. The AGES-Reykjavik study originates from the Reykjavik study established in 1967, which consisted of 30,795 randomly sampled men and women born 1907-1935. This large cohort equalled roughly 35% of this age-specific population in Iceland. The AGES-Reykjavik cohort was randomly sampled from the 11,549 individuals still alive when examinations began and is thought to represent the study population fairly well. The participants in the present study were heavier, had less fat free (FFM) mass, more fat mass (FAT) and a higher BMI. The weight and amount of FAT may possibly be related to our study group being slightly younger than the average participant in the AGES study, as aging is commonly accompanied by weight loss [27,28]. With respect to the lower FFM of our participants, the fact that they signed up voluntarily to participate in the IceProQualita study, which included supervised exercise three times per week, may indicate that they themselves felt their physical fitness needed improvement, and that their weight should be better managed. This is further emphasized by the fact that our participants spent less time walking than the AGES participants, indicating that they were less physically active.

In spite of statistical significance for selected variables between participants of the present study and the AGES study, these differences are not extensive. Therefore, our study sample is still thought to represent the AGES group adequately for the purposes of validation.

Weighed food records are generally perceived as a good measure of food intake [11,12], and have the least correlated errors with food frequency questionnaires [12]. However, day-to-day variation can be great and even greater for individual food items than for nutrient intake [11]. Hence, a longer period of food recording, or repeated recordings, would have been needed in the present study to find correlation to certain answers of the AGES-FFQ.

Nonetheless, a majority of the questions in the AGES-FFQ had an acceptable correlation (r = 0.3-0.7) and may therefore be used to rank individuals according to intake. Questions with lower or insignificant correlation, such as on fish and meat consumption, should not be ruled out or considered invalid without further assessment, as the validity of certain questions is likely to be underestimated rather than exaggerated. However, the same applies here as in the previous study on the AGES-FFQ on midlife diet, that is, that even though the AGES-FFQ on present diet is able to rank individuals according to their intake of several important food groups, one should always be aware of the limitations of the method and the different results seen for different food items. It should also be noted that the AGES-FFQ is only appropriate for ranking individuals according to level of intake of selected foods and food groups, and not for assessing total food intake, energy or nutrients.

## Conclusion

One of the most important factors related to health and quality of life in old age is nutrition [29-32]. It is also a factor we largely control ourselves and can therefore adjust to enhance our likelihood of successful aging [9,32,33]. Studies have shown that even in old age, adherence to a healthy diet or changes in lifestyle to improve health can affect risk factors for chronic diseases [34-36]. While some conditions develop over many years, others may occur within weeks [37].

It is our conclusion that the AGES-FFQ on present diet may be used to rank individuals according to consumption of several important foods and food groups. As a result the extensive data gathered from the elderly participants of the AGES-Reykjavik Study may be available for studies of associations between diet and health-related variables in this large epidemiological study.

## Abbreviations

FFQ: Food Frequency Questionnaire; AGES (Reykjavik Study): Age Gene/Environment Susceptibility (Reykjavik Study); Y: year; MMSE: Mini Mental State Examination; BMI: Body mass index; FFM: Fat free mass; FAT: Fat mass.

## Competing interests

The authors declare that they have no competing interests.

## Authors' contributions

TE gathered dietary data, handled statistical analysis and interpretation of data, and drafted the manuscript. IT, IG and LS participated in the conception and design of the study and revision of the manuscript. LS contributed to the design of the food frequency questionnaire. All authors have read and approved the final manuscript.

## Authors' information

TE is a PhD student and has assessed the validity of questions in the AGES-FFQ on midlife diet in a previous paper. LS, currently a professor of nutrition at the University of Iceland, was formerly director of the Icelandic Nutrition Council, and in that capacity managed two national nutrition surveys as well as other programs of the council, including co-operative projects with the Icelandic Heart Association. IT is a professor of nutrition and head of the Unit for Nutrition Research, University of Iceland and Landspitali National University Hospital. IT has long experience in co-ordinating studies e.g., validation studies, and is a principal investigator of the IceProqualita study. IG, a professor of nutrition at the University of Iceland, has co-ordinated and participated in several dietary surveys, some of them involving validation of methods. LS and IT supervise TE's PhD research project and IG is a member of TE's PhD committee.

## Supplementary Material

Additional file 1 **Gender-specific portions (g) were estimated taking into account actual intake in grams and eating occasions from food diaries, as well as predetermined portion sizes used in a previous validation study of questions on midlife diet**. Click here for file

Additional file 2 **Shows the results from the nonparametric Jonckheere-Terpstra test for trend performed to assess whether the AGES-FFQ ranked mean intake from the food record in an anticipated, graded order**. Click here for file

Additional file 3 **Shows the results from Pearson Chi-Square and Kendall's tau-b tests performed to further assess the association between the two different dietary assessment methods**. Click here for file
