# Supplementary material for: Assessing validity of a short food frequency questionnaire on present dietary intake of elderly Icelanders
Source: Nutr J. 2012 Mar 13;11:12. doi: 10.1186/1475-2891-11-12 (PMC3349496; doi:10.1186/1475-2891-11-12)
Supplement: Additional file 1 — Gender-specific portions (g) were estimated taking into account actual intake in grams and eating occasions from food diaries, as well as predetermined portion sizes used in a previous validation study of questions on midlife diet. [file 1475-2891-11-12-S1.DOC]

**Additional file 1.** Gender-specific portion sizes

|  |  | |  | |  |
| --- | --- | --- | --- | --- | --- |
|  | | **Men** | | **Women** | |
| Meat | | 200 | | 135 | |
| Fish | | 170 | | 130 | |
| Fish toppings | | 45 | | 70 | |
| Potatoes | | 110 | | 85 | |
| Fresh fruit | | 110 | | 120 | |
| Blood/liver sausage | | 80 | | 60 | |
| Rye bread/flatbread | | 50 | | 60 | |
| Whole wheat bread | | 50 | | 45 | |
| Oatmeal/muesli | | 190 | | 150 | |
| Cocked vegetables | | 90 | | 90 | |
| Fresh vegetables | | 90 | | 80 | |
| Cakes and cookies | | 70 | | 60 | |
| Candy | | 35 | | 30 | |
| Dairy products | | 205 | | 170 | |
| Milk | | 165 | | 135 | |
| Pure fruit juice | | 160 | | 160 | |
| Soft drink and sweet juice | | 345 | | 225 | |
| Fish liver oil | | 5.6 | | 7.2 | |
| Coffee | | 210 | | 195 | |
| Tea | | 220 | | 240 | |
| Sugar | | 5 | | 5 | |

Gender-specific portions (g) were estimated taking into account actual intake in grams and eating occasions from the food diaries, as well as predetermined portion sizes used in our previous validation study of questions on midlife diet [21].
