# Supplementary material for: Assessing validity of a short food frequency questionnaire on present dietary intake of elderly Icelanders
Source: Nutr J. 2012 Mar 13;11:12. doi: 10.1186/1475-2891-11-12 (PMC3349496; doi:10.1186/1475-2891-11-12)
Supplement: Additional file 2 — Shows the results from the nonparametric Jonckheere-Terpstra test for trend performed to assess whether the AGES-FFQ ranked mean intake from the food record in an anticipated, graded order. [file 1475-2891-11-12-S2.DOC]

**Table 5a Average consumption in g/d (sd) depending on answers given in the AGES-FFQ**

|  | **Frequency of consumption** | | | | |  |
| --- | --- | --- | --- | --- | --- | --- |
| **Men** | **1^*^** | **2**^†^ | **3**^‡^ | **4**^§^ | **5**^¶^ | **trend^**^** |
|  |  |  |  |  |  |  |
| Meat | 73 (59) | 93 (62) | 108 (91) | 207 (74) |  | 0.2999 |
|  | n = 2 | n = 19 | n = 28 | n = 4 | n = 0 |  |
| Fish | 0 (.) | 76 (41) | 93 (69) | 156 (94) |  | 0.0933 |
|  | n = 1 | n = 15 | n = 32 | n = 5 | n = 0 |  |
| Fish topping/salad | 1 (4) | 7 (12) | 14 (23) | 0 (.) | 5 (8) | 0.0245 |
|  | n = 21 | n = 19 | n = 8 | n = 1 | n =3 |  |
| Potatoes |  | 54 (63) | 52 (22) | 87 (56) | 113 (51) | 0.0007 |
|  | n = 0 | n = 6 | n = 6 | n = 16 | n = 25 |  |
| Fresh fruit | 0 (.) | 40 (63) | 60 (49) | 165 (93) | 153 (95) | <0.0001 |
|  | n = 2 | n = 8 | n = 15 | n = 9 | n = 18 |  |
| Blood/liver sausage | 3 (13) | 7 (22) | 0 (.) | 0 (.) | 14 (.) | 0.5342 |
|  | n = 38 | n = 12 | n = 1 | n =1 | n = 1 |  |
| Rye bread | 7 (12) | 22 (23) | 23 (21) | 36 (28) | 26 (39) | 0.1083 |
|  | n = 9 | n = 14 | n = 17 | n = 4 | n = 8 |  |
| Whole wheat bread | 15 (21) | 40 (28) | 48 (42) | 41 (21) | 50 (28) | 0.0358 |
|  | n = 2 | n = 6 | n = 9 | n = 8 | n = 27 |  |
| Oatmeal/muesli | 6 (24) | 51 (61) | 40 (52) | 83 (63) | 115 (128) | 0.0014 |
|  | n = 14 | n = 8 | n = 10 | n = 4 | n = 17 |  |
| Cooked vegetables | 33 (58) | 38 (45) | 57 (63) | 33 (28) | 92 (.) | 0.1120 |
|  | n = 10 | n = 26 | n = 11 | n = 4 | n = 1 |  |
| Fresh vegetables | 10 (15) | 57 (57) | 47 (56) | 66 (29) | 102 (78) | 0.0112 |
|  | n = 7 | n = 20 | n = 15 | n = 5 | n = 6 |  |
| Cakes and cookies | 38 (40) | 42 (39) | 55 (54) | 116 (95) | 112 (86) | 0.0117 |
|  | n = 12 | n = 14 | n = 10 | n = 6 | n = 10 |  |
| Candy | 0 (1) | 9 (15) | 12 (31) | 3 (4) |  | 0.0092 |
|  | n = 24 | n = 15 | n = 10 | n = 4 | n = 0 |  |
| Dairy products | 20 (49) | 64 (62) | 88 (54) | 80 (44) | 249 (145) | <0.0001 |
|  | n = 7 | n = 14 | n = 15 | n = 7 | n = 10 |  |
| Milk | 90 (103) | 184 (105) | 209 (232) | 132 (115) | 285 (222) | 0.0014 |
|  | n = 20 | n = 5 | n = 7 | n = 2 | n = 19 |  |
| Pure fruit juice | 8 (30) | 20 (60) | 45 (86) | 129 (155) | 126 (114) | 0.0003 |
|  | n = 24 | n = 9 | n = 7 | n = 3 | n = 9 |  |
| Soft drink/sweet juice | 65 (218) | 49 ( 61) | 113 (58) | 60 (70) | 0 (.) | 0.0731 |
|  | n = 34 | n = 9 | n = 4 | n = 4 | n = 1 |  |
| Cod liver oil | 0 (0) | 0 (.) | 6 (.) | 0.1 (0.2) | 3 (3) | 0.0003 |
|  | n = 14 | n = 1 | n = 1 | n = 3 | n = 34 |  |
| Coffee^††^ | 122 (95) | 298 (169) | 450 (211) | 529 (310) | 792 (227) | <0.0001 |
|  | n = 8 | n = 14 | n = 23 | n = 5 | n = 3 |  |
| Tea^††^ | 30 (58) | 154 (114) | 206 (123) |  |  | <0.0001 |
|  | n = 39 | n = 11 | n = 3 | n=0 | n=0 |  |
| Sugar^††^ | 1 (4) | 18 (21) | 0 (.) |  |  | <0.0001 |
|  | n = 46 | n = 6 | n = 1 | n=0 | n=0 |  |

**Table 5b Average consumption in g/d (sd) depending on answers given in the AGES-FFQ**

|  | **Frequency of consumption** | | | | |  |
| --- | --- | --- | --- | --- | --- | --- |
| **Women** | **1^*^** | **2**^†^ | **3**^‡^ | **4**^§^ | **5**^¶^ | **trend^**^** |
|  |  |  |  |  |  |  |
| Meat | 52 (68) | 73 (48) | 70 (41) | 92 (37) | 0 | 0.2364 |
|  | n = 6 | n = 36 | n = 30 | n = 3 | n = 0 |  |
| Fish | 16 (23) | 71 (48) | 68 (40) | 57 (49) |  | 0.8744 |
|  | n = 2 | n = 28 | n = 37 | n = 8 | n = 0 |  |
| Fish topping/salad | 3 (10) | 7 (16) | 12 (13) | 45 (7) | 36 (1) | 0.0007 |
|  | n = 45 | n = 22 | n = 4 | n = 2 | n = 2 |  |
| Potatoes | 72 (61) | 74 (34) | 54 (39) | 73 (34) | 58 (32) | 0.6313 |
|  | n = 3 | n = 8 | n = 23 | n = 18 | n = 23 |  |
| Fresh fruit | 0 (0) | 78 (45) | 100 (47) | 111 (46) | 175 (122) | 0.0019 |
|  | n = 1 | n = 5 | n = 6 | n = 13 | n = 50 |  |
| Blood/liver sausage | 2 (10) | 7 (16) | 16 (17) |  |  | 0.0010 |
|  | n = 57 | n = 15 | n = 3 | n = 0 | n = 0 |  |
| Rye bread | 7 (16) | 4 (7) | 28 (28) | 18 (21) | 23 (16) | 0.0002 |
|  | n = 16 | n = 18 | n = 20 | n = 8 | n = 13 |  |
| Whole wheat bread | 26 (46) | 51 (46) | 32 (20) | 34 (37) | 47 (29) | 0.0119 |
|  | n = 5 | n = 8 | n = 14 | n = 9 | n = 39 |  |
| Oatmeal/muesli | 12 (26) | 27 (34) | 62 (50) | 198 (137) | 92 (67) | <0.0001 |
|  | n = 14 | n = 12 | n = 16 | n = 3 | n = 28 |  |
| Cooked vegetables | 44 (43) | 24 (32) | 28 (34) | 41 (28) | 73 (56) | 0.0486 |
|  | n = 10 | n = 25 | n = 24 | n = 6 | n = 10 |  |
| Fresh vegetables | 20 (21) | 50 (39) | 46 (41) | 90 (55) | 93 (64) | 0.0029 |
|  | n = 5 | n = 19 | n = 21 | n = 15 | n = 15 |  |
| Cake and cookie | 36 (45) | 49 (31) | 39 (55) | 42 (33) | 71 (49) | 0.1214 |
|  | n = 13 | n = 23 | n = 17 | n = 6 | n = 16 |  |
| Candy | 1 (2) | 9 (21) | 11 (15) | 7 (5) | 13 (11) | 0.0001 |
|  | n = 24 | n = 24 | n = 15 | n = 4 | n = 8 |  |
| Dairy products | 22 (37) | 88 (76) | 123 (92) | 114 (74) | 159 (83) | <0.0001 |
|  | n = 10 | n = 19 | n = 13 | n = 15 | n = 18 |  |
| Milk | 95 (132) | 107 (80) | 126 (25) | 8 (11) | 225 (123) | <0.0001 |
|  | n = 29 | n = 11 | n = 4 | n = 2 | n = 29 |  |
| Pure fruit juice | 3 (8) | 39 (71) | 26 (47) | 73 (86) | 101 (93) | 0.0001 |
|  | n = 25 | n = 15 | n = 10 | n = 9 | n = 16 |  |
| Soft drink/sweet juice | 32 (71) | 45 (75) | 108 (74) |  |  | 0.0807 |
|  | n = 61 | n = 10 | n = 4 | n = 0 | n = 0 |  |
| Cod liver oil | 0.2 (0.5) |  | 0 (.) | 11 (13) | 5 (5) | 0.0039 |
|  | n = 11 | n = 0 | n = 1 | n = 4 | n = 56 |  |
| Coffee^††^ | 125 (105) | 307 (164) | 376 (214) | 522 (350) |  | 0.0002 |
|  | n = 11 | n = 30 | n = 30 | n = 4 | n = 0 |  |
| Tea^††^ | 23 (51) | 189 (169) | 249 (294) | 133 (.) |  | <0.0001 |
|  | n = 36 | n = 30 | n = 8 | n = 1 | n = 0 |  |
| Sugar^††^ | 1 (2) | 5 (9) |  |  |  | 0.0252 |
|  | n = 71 | n = 3 | n = 0 | n = 0 | n = 0 |  |

***** 1: never or less than once a week

† 2: 1-2 times a week

‡ 3: 3-4 times a week

§ 4: 5-6 times a week

¶ 5: daily or more than once a day

** Jonckheere-Terpstra trend test

†† Consumption in times/portions per day in stead of per week
