# Supplementary material for: Assessing validity of a short food frequency questionnaire on present dietary intake of elderly Icelanders
Source: Nutr J. 2012 Mar 13;11:12. doi: 10.1186/1475-2891-11-12 (PMC3349496; doi:10.1186/1475-2891-11-12)
Supplement: Additional file 3 — Shows the results from Pearson Chi-Square and Kendall's tau-b tests performed to further assess the association between the two different dietary assessment methods. [file 1475-2891-11-12-S3.DOC]

**Additional file 3. Association between the two methods (Chi-Square or Kendall’s tau-b)**

|  | Men | Women |
| --- | --- | --- |
|  | p-value | p-value |
| Meat^*^ | 0.389 | 0.762 |
| Fish^*^ | 0.226 | 0.449 |
| Fish toppings^*^ | 0.034 | 0.008 |
| Potatoes^**^ | <0.001 | 0.370 |
| Fresh fruits^**^ | <0.001 | 0.007 |
| Blood/liver sausage^*^ | 0.488 | 0.001 |
| Rye bread/flatbread^**^ | 0.084 | <0.001 |
| Whole-wheat bread^**^ | 0.226 | 0.005 |
| Oatmeal/muesli^**^ | 0.015 | <0.001 |
| Cooked vegetables^**^ | 0.150 | 0.226 |
| Raw vegetables^**^ | 0.204 | 0.001 |
| Cakes and cookies^**^ | 0.004 | 0.226 |
| Candy^*^ | 0.257 | 0.003 |
| Dairy products^**^ | <0.001 | <0.001 |
| Milk^**^ | 0.001 | <0.001 |
| Pure fruit juice^*^ | <0.001 | 0.002 |
| Soft drink and sweet juice^*^ | 0.105 | 0.170 |
| Cod liver oil^*^ | <0.001 | <0.001 |
| Coffee^¥*^ | <0.001 | 0.009 |
| Tea^¥*^ | <0.001 | <0.001 |
| Sugar in coffee/tea^¥*^ | 0.001 | 0.015 |

* Pearson Chi-Square

** Kendall’s tau-b

¥ Daily consumption

Data from the AGES-FFQ was split into 2-4 groups depending on distribution of answers from each question, data from food record was split into comparable groups. Kendall’s tau-b rank correlation coefficient or Chi-Square test was used to examine association between the two methods.
